# How Does Employees’ Narcissism Influence Organizational Commitment? The Role of Perceived Supervisor Support and Abusive Supervision

**DOI:** 10.3389/fpsyg.2022.910739

**Published:** 2022-05-25

**Authors:** Boxiang Yu, Yong Meng, Chaoping Li, Dege Liu

**Affiliations:** ^1^Management Institute, Xinxiang Medical University, Xinxiang, China; ^2^School of Public Administration and Policy, Renmin University of China, Beijing, China; ^3^School of Management, Guangzhou University, Guangzhou, China

**Keywords:** employees’ narcissism, perceived supervisor support, organizational commitment, self-value threat, abusive supervision

## Abstract

Narcissism has an important influence on employees’ attitudes and behavior. However, research on the mechanism of this process is still relatively scarce. Based on the conservation of resources (COR) theory, this study constructed a conceptual model of the relationship between narcissism and organizational commitment and explored the role of perceived supervisor support and abusive supervision in this process. Data were collected in three waves from 288 participants through an online data collection platform in China. The results indicated that employee narcissism negatively predicts organizational commitment, and this process is mediated by perceived supervisor support. We also discuss the moderating role of abusive supervisors on perceived supervisor support, confirming that external self-value threat affects perceived support of narcissistic individuals. These results contribute to our understanding of the role of narcissism in organizations.

## Introduction

As a kind of “dark personality” in the traditional sense, narcissism (i.e., a grandiose sense of self and expectations of special treatment from others) has been extensively researched by organizational behaviorists in recent years (Grijalva et al., 2014; Grijalva and Newman, 2015; Wang et al., 2020). However, a systematic review of the existing narcissism literature related to the organizational context indicated that most studies have focused on narcissism and its negative behavioral consequences in employees (Grijalva and Newman, 2015), while relatively few studies focused on employees’ job-related attitudes (Campbell et al., 2010). Organizational commitment is one of the core factors of employee’s attitudes in the workplace (Schumacher et al., 2016). Explaining the relationship between organizational commitment and narcissism can provide necessary theoretical support for understanding narcissism’s behavioral consequences and work attitude in the organization. However, the conclusions of existing studies on the relationship between narcissism and organizational commitment are inconsistent. Michel and Bowling (2013) research showed that narcissism helps enhance an individual’s organizational commitment, while Choi (2019) analyzed perceived organizational support as a mediator of the relationship between narcissism and organizational commitment and drew an opposite conclusion. These contradictory conclusions suggest that a more complex psychological mechanism between narcissism and organizational commitment may be involved.

From the perspective of resource conservation, Hobfoll (1989) takes personal resources as the core concept to construct the generation and coping model of individual stress in different situations. When people perceive the depletion of resources or predict that their investment in resources will not get the expected return on resources, the sense of pressure will occur accordingly (Hobfoll, 1989), and a series of negative results will be produced [e.g., Halbesleben and Wheeler (2015) and Kronenwett and Rigotti (2019)]. Because of the unique egocentric point of view, narcissistic employees have an increased demand for resources and are more sensitive to resource consumption (Zhou et al., 2020). Meanwhile, they also have difficulty maintaining trust and intimate relationships in interpersonal communication (Fang et al., 2021a). Considering that supervisors in an organization are responsible for providing resource supplementation for individuals and interpersonal emotional support (Gordon et al., 2019), we believe that narcissistic employees are affected by their characteristics, and it is difficult to get effective resource supplements from supervisors and the obstruction of access to “supervisor support” will have a significant impact on individual work attitudes (Gordon et al., 2019).

Hobfoll et al. (2018) believe that the ability to obtain and use resources from the situation is of the same importance as the individual resource reserve, which constitutes the core element for coping with pressure and resource expansion. Considering the universality of personality influence (Mischel and Shoda, 1995), we reasonably speculate that individuals with different narcissistic degrees in the same situation will have different resource acquisition cognition. Researchers know abusive supervision as a way of management by putting pressure on the self-value of subordinates (Liu et al., 2012). As a subjective assessment of supervisor hostility (excluding physical contact) rather than an objective quantification (Tepper et al., 2017), abusive supervision, to a certain extent, reflects the employee’s assessment of situational stress derived from superiors (Ganster and Rosen, 2013; Tepper et al., 2017). Given the stress sensitivity of narcissistic individuals and their need for a unique sense of self-superiority (Campbell et al., 2010), we focused on abusive supervision as a stressor in organizational contexts to explore how the interaction of narcissism with self-value threats influences employees’ perceptions of supervisor support. Discuss the possible influence of external stress factors on narcissism and perceived supervisor support relationship.

At present, there is no research on the possible impact of abusive supervision style on narcissistic employees’ resource cognition as an external self-value threat (Liu et al., 2012). At the same time, from the perspective of resource acquisition of conservation of resources (COR) theory, there are few studies on the impact of narcissism on resource acquisition perception by using supervisor support as a resource acquisition channel (Choi, 2019). Narcissistic employees have a unique cognitive model of resource acquisition and interpersonal communication (Campbell et al., 2010; Huang et al., 2020). The discussion from the perspective of resource supplement is helpful for us to understand better the internal psychological mechanism that narcissism influences organizational commitment (Choi, 2019). With the improvement of material living standards, the degree of narcissism has shown a significant upward trend in the new generation (Hamamura, 2017). Understanding the cognitive and behavioral patterns of narcissists in the organization will help to optimize better the organizational management system and practice (Mao et al., 2021). Therefore, this study focuses on narcissistic employees’ perceived supervisor support to explore how narcissism affects individuals’ organizational commitment and discusses the impact of self-value threats (abusive supervision) on this process.

### Narcissism and Organizational Commitment

In related research, narcissism is widely defined as a disposition of grandiosity, self-love, and inflated self-views (Campbell et al., 2010). It is characterized by a desire for power and admiration in relationships, a lack of empathy, a perception of entitlement, and the exploitation of others (Campbell and Campbell, 2009). Furthermore, the most accepted definition of organizational commitment is a psychological phenomenon of the degree of psychological bond between the employee and the organization (Allen and Meyer, 1990). Precisely, organizational commitment reflects the employee’s willingness to be a member of the organization, the psychological sense of belonging to the organization, and the degree of willingness to devote energy and resources to the organization’s development (Allen and Meyer, 1996). Organizational commitment is also regarded as the internal reflection of employees’ psychological contract with the organization; that is, their sense of mutual obligation with it (Rousseau et al., 2016).

Based on the existing literature, Campbell et al. (2010) discussed the relationship between narcissism and organizational perception regarding organizational justice perception and psychological contract violation. Resource conservation theory provides an appropriate explanation mechanism for narcissists’ negative emotional cognition of the organization. One of the core attributes of narcissism is the sense of psychological entitlement. Driven by this motivation, narcissistic employees believe that they should obtain more resources than other colleagues (Campbell et al., 2010). When narcissists think that the energy, time, and knowledge resources they put into the organization are not “reciprocally” rewarded, the sense of injustice with the organization tends to increase (Hobfoll et al., 2018). Even if the organization treats employees equally, narcissistic employees are more likely to be dissatisfied with their resource feedback from the organization, driven by their psychological sense of privilege (Paulhus and Williams, 2002). The psychological gap of resource expectations will be transformed into internal psychological pressure and then manifested as negative emotions and attitudes toward the organization (Hobfoll, 1989).

On the other hand, due to the influence of self-serving bias, narcissists tend to exaggerate their abilities and importance to the organization (Carlson et al., 2011). Job skills and abilities as the core resource types for individuals to invest in the organization (Hobfoll et al., 2018). Too high perception of overqualification will lead to employees’ negative emotions toward the organization and aggravate the individual’s intention to leave (Harari et al., 2017). Narcissists “work for themselves” (Campbell et al., 2010) and are more likely to perceive that the organization does not recognize their talents and violates the psychological contract of resource interaction between organization and individual (Rousseau, 1989). Considering that the psychological contract is the basis of employees’ organizational commitment (Rousseau et al., 2016), we propose the following hypothesis:

H1. Narcissism is negatively related to organizational commitment.

### The Mediating Role of Perceived Supervisor Support

Kottke and Sharafinski (1988) put forward the concept of perceived supervisor support (PSS), which refers to employees’ overall view that supervisors attach importance to their values and well-being. As one of the most important social support systems in the organization for employees, PSS dramatically affects the relationship between employees and organizations (Gordon et al., 2019).

The relationship between PSS and organizational commitment has been confirmed by previous study (Talukder, 2019), but no study has discussed the relationship between narcissism and PSS. Based on the perspective of narcissistic subordinates, people with narcissism are characterized as having high psychological privilege, lack of empathy, an extreme need for self-enhancement, and a sense of superiority (Campbell et al., 2010), which lead to narcissists’ lack of intimacy and difficulty maintaining trust in others (Burgmer et al., 2019). Additionally, Narcissists pursue organizational status that is superior to others, and their desire for promotion will cause narcissists to regard their superiors who are higher than their organizational hierarchy as an obstacle to promotion (Campbell and Campbell, 2009). Narcissists’ superficial experience of intimacy and hostility to their superiors make it difficult for them to perceive adequate resource support from the supervisor (Campbell et al., 2010; Zhou et al., 2020). Furthermore, for the supervisor of narcissistic employees, through the way supervisors evaluate employees, Blair et al. (2008) found that most supervisors have a negative attitude toward narcissistic employees, and they believe that narcissistic subordinates in the team will have a destructive impact on the daily management and norms of the team. Considering the negative correlation between narcissism and agreeableness (i.e., a trait associated with cooperation), we think supervisors may be more likely to support non-narcissistic subordinates than narcissistic employees.

Combined with the COR theory, we attempt to explain the path via which narcissism affects organizational commitment by perceiving supervisor support from the perspective of resource conservation. The primary motivation of individual action is to obtain resource support while avoiding the depletion of resources (Halbesleben et al., 2014; Hobfoll et al., 2018). Narcissists maintain a sense of superiority and psychological privilege in the organization (Campbell et al., 2010) and consume many psychological resources to maintain an exaggerated self-perception (Choi, 2019). Meanwhile, as one of the core support systems in the organization, supervisor support is the primary source of resource supplementation for employees (Skiba and Wildman, 2019). Narcissistic employees’ distrust and hostile perception of leaders make it difficult to obtain effective resource supplementation from supervisor support.

Furthermore, while narcissists maintain high resource input awareness of the organization, the lack of adequate perception of supervisor support will cause narcissists to fall into the spiral of loss (Hobfoll et al., 2018). Narcissistic employees cannot effectively prevent the loss of psychological resources, nor can they get adequate resource compensation from the environment, which will lead to individual perception of the increasing pressure from the environment (Hobfoll et al., 2018). Over an extended period, the psychological state of resource exhaustion will inevitably lead to a series of negative attitudes and behavioral outcomes (Zhang et al., 2020), which will reduce employees’ organizational commitment. Combining the above arguments, we propose the following hypothesis:

H2. PSS mediates the effects of narcissism on organization commitment.

### Abusive Supervision as a Moderator

Abusive supervision refers to hostile verbal or non-verbal behavior toward a subordinate but does not include physical contact (Tepper et al., 2008). In consideration of the attributes and characteristics of narcissistic employees, this study aims to explore whether the external pressure experienced by narcissistic individuals will lead to distorted perceptions of external resource support. The core behavioral goal of narcissists is to maintain their sense of superiority in their environment (Schyns et al., 2019) while maintaining positive self-awareness (Campbell et al., 2010). In abusive leadership, which belittles one’s value and denies personal ability (Tepper et al., 2008), employees will face tremendous physical and psychological pressure and negatively perceive their working ability and self-value (Tepper et al., 2017). Unlike the general group’s response to supervision hostility behavior that threatens self-value, we speculate that narcissistic employees will use cognitive regulatory strategies to reduce the threat of external factors to maintain their sense of superiority (Campbell et al., 2010).

Combined with the previous discussion on the relationship between narcissism and PSS, we infer that with greater narcissism, PSS decreases under the condition of having a minimally abusive supervisor. In other words, narcissism is negatively correlated with PSS in general. However, under the condition of a highly abusive supervisor, the denial and blame faced by employees will cause them to fall into self-doubt (Liu et al., 2012). Thus, employees with low narcissism will think that supervisors do not value them and correspondingly perceive a lower sense of supervisor support (Zhang and Liao, 2015). Due to that narcissists’ need to maintain a sense of self-superiority (Campbell et al., 2010), and self-regulation strategies are one of the main components of narcissism (Campbell et al., 2010). We infer that high narcissists will experience cognitive dissonance; that is, they will attempt to reduce cognitive threats by changing their cognition to alleviate the stressful factors they face (Harmon-Jones and Mills, 2019). Therefore, when faced with highly abusive leaders, we speculate that narcissistic employees will distort this abusive behavior, interpreting it as a sign that the supervisor recognizes their value and thinks they obtain more support from the supervisor. Based on this, we propose the following hypothesis:

H3a. Abusive supervision moderates the negative relationship between narcissism and PSS.

**FIGURE 1:**
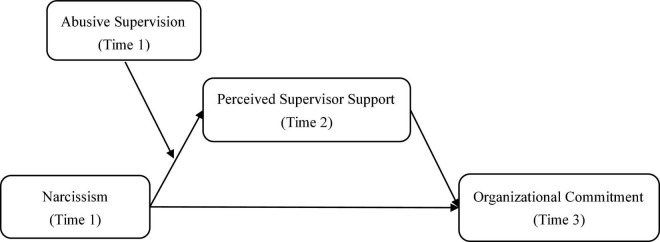
Proposed model.

Combined with hypotheses H2 and H3a, we further propose a first stage moderated mediation model with abusive supervision (Figure 1) to test whether abusive supervision can regulate the indirect effect of narcissism on organizational commitment. Therefore, we propose the following:

H3b. Abusive supervision moderates the indirect effect of narcissism on organizational commitment via PSS.

### Present Study

To verify these research hypotheses, we plan to conduct a 3-month data survey in China (2021.10–2021.12). Considering the negative impact of the pandemic on on-site research, we decided to conduct online research by recruiting qualified participants through the Internet [e.g., Meekaewkunchorn et al. (2021)]. After screening qualified adult full-time employees, we plan to complete the survey at three-time points, 2 weeks apart each time. The second part of the article will briefly introduce the data collection methods and research tools we used. In the third part, we carried out a series of analyses of the collected data to test our research hypothesis. The contributions and shortcomings of the article will be discussed in the last part.

## Materials and Methods

### Participants

We recruited participants through Tencent Survey,^1^ a third-party online data collection platform designed for surveys, and obtained 441 respondents. To reduce the impact of biases from common sources, we divided data collection into three phases, each with an interval of 2 weeks. The entire survey was conducted online, with each participant regularly receiving links to research from The Tencent survey and being reminded to respond. At time 1, 441 participants completed narcissism, abusive supervision, and demographics measures, and each participant was rewarded 2.5 yuan (about 0.39 dollars). At time 2, 372 participants (response rate = 90.5%) reported their PSS. Each participant was rewarded 7.5 yuan (about 1.17 dollars). At time 3, 345 participants (response rate = 92.7%), reported their organizational commitment. Each participant was rewarded 10 yuan (about 1.56 dollars). All participant data were coded and matched across the different stages. Participants were assured anonymity of their responses and that their data would remain confidential.

To ensure the effectiveness of online data collection, we further screened the data by examining the attention check items (participants were asked to choose “strongly agree” on three items for each survey) and the response time (Porter et al., 2019). Finally, we obtained a valid sample of 288 employees. Of the participants, 145 were men (50.3%), and the mean age of participants was 28.9 years (SD = 5.95, range = 18–58). In terms of education level, 33.7% were below undergraduate level (*n* = 97), 57.3% had an undergraduate degree (*n* = 165), and 9.0% had a master’s degree or above (*n* = 26). The average working tenure was 6.55 years (SD = 5.36), with an average of 3.88 (SD = 4.22) years in the current organization.

### Measures

All the scales used were validated in the Chinese context, and participants were asked to respond on a five-point Likert scale in these measurements, ranging from “1 = strongly disagree” to “5 = strongly agree.” Participants were asked to indicate their agreement with the statement according to their understanding.

#### Narcissism

We measured employee narcissism with a six-item scale developed by Hochwarter and Thompson (2012). This scale was developed to measure supervisor narcissism, and we adapted it to measure employee narcissism (e.g., “I am a very self-centered person”). This scale has good reliability in the Chinese context (α = 0.89; Chen et al., 2018). And in this study, the reliability of the total score was 0.80 (α) and 0.81 (ω).

#### Abusive Supervision

We adopted a ten-item scale developed by Tepper (2000) and adapted by Aryee et al. (2008) to measure abusive supervision (e.g., “My immediate supervisor makes negative comments about me to others”). This scale has a Chinese version and has been shown to have a good reliability coefficient (α = 0.88). The reliability coefficient in our study was 0.89 (α) and 0.90 (ω).

#### Perceived Supervisor Support

This factor was measured using the four-item scale adapted by Rhoades et al. (2001). A sample item is “My supervisor cares about my well-being.” The psychometric properties of this scale among Chinese samples have been verified by previous studies [α = 0.92; Huo and Jiang (2021)]. In this study, the reliability of the perceived supervisor support scale was 0.80 (α) and 0.81 (ω).

#### Organizational Commitment

We used Allen and Meyer (1996) six-item scale to measure organizational commitment (e.g., “I am delighted to work in this unit in the future”). This scale is widely used in organizational research in China and has good psychometric properties [α = 0.79; Li et al. (2006)]. Cronbach’s alpha coefficient for this scale in our study was 0.91. The omega coefficient has the same value.

#### Control Variables

Researchers have suggested that gender (0 = male, 1 = female), age, educational level (1 = high school and below, 2 = junior college degree, 3 = undergraduate degree, 4 = master’s degree or above), and organizational tenure may affect employees’ organizational commitment (Mathieu et al., 1990). Therefore, we controlled these variables in all analyses with reference to previous studies (e.g., Ling, 2013; Jiang et al., 2015).

## Results

### Preliminary Analysis

Table 1 presents the descriptive statistical information of the relevant variables in this study, including the correlation coefficients, reliability coefficients (α and ω) of the measure, means, standard deviations, composite reliabilities (CR), and average variance extracted (AVE). As shown in Table 1, the CR values of all variables are higher than 0.6, and the AVE values meet the acceptable criteria of being >0.4 (Bagozzi and Yi, 1988), indicating an acceptable convergent validity. All four constructs measured in this study have good internal consistency coefficients (great than 0.8). At the same time, the square root of AVE of each variable is greater than the correlation coefficient between the variables, and the discriminant validity of the measurement model was supported.

**TABLE 1:** Descriptive statistics and correlations.

Variables	Mean	SD	α/ω	CR	AVE	1	2	3	4	5	6	7	8
Gender	—	—	—	—	—	—							
Age	28.9	5.95	—	—	—	–0.140*	—						
Education	2.69	0.72	—	—	—	0.000	0.239***	—					
Tenure	6.55	5.36	—	—	—	–0.090	0.648***	0.110	—				
NA	2.04	0.81	0.80/0.81	0.81	0.41	–0.286***	–0.198***	0.070	–0.147*	(0.64)			
AS	2.21	0.83	0.89/0.90	0.90	0.50	–0.136*	–0.087	–0.010	–0.040	0.376***	(0.70)		
PSS	3.65	0.83	0.80/0.81	0.81	0.52	0.040	0.116*	0.000	0.070	–0.193**	–0.515***	(0.72)	
OC	3.56	0.99	0.91/0.91	0.91	0.64	–0.073	0.186**	0.068	0.216***	–0.129*	–0.355***	0.454***	(0.80)

N = 288. *p < 0.05, **p < 0.01, ***p < 0.001. Gender: Male = 1, female = 2. NA, narcissism; AS, abusive supervision; PSS, perceived supervisor support; OC, organizational commitment; CR, composite reliability; AVE, average variance extracted, squared roots of AVEs are presented in the brackets along the diagonal.

We conducted a series of confirmatory factor analyses using package “lavaan” (Rosseel, 2012) in R 4.1.0 to test the measurement model. As shown in Table 2, the four-factor model fit the data best: χ^2^_(293)_ = 677, *p* < 0.001, confirmatory fit index = 0.901, root mean square error of approximation = 0.067, 90% confidence interval (CI) [0.060, 0.074], and standardized root mean squared residual = 0.058, which suggested adequate discriminant validity of the measurement model. It is worth noting that considering that both AS and PSS are derived from employees’ evaluation of their direct managers, to verify the differentiation of these two variables in the measurement structure, we first merge the items of the two scales to build a competition model. This model had poor fit to the data: χ^2^_(272)_ = 747, *p* < 0.001, confirmatory fit index = 0.869, root mean square error of approximation = 0.078, 90% confidence interval (CI) [0.071, 0.084], and standardized root mean squared residual = 0.070, the CFI and RMSEA change was much greater than 0.01 (△CFI = 0.03, △RMSEA = 0.011), indicating that this change between four-factor and three-factor model was significant (Cheung and Rensvold, 2002). The effectiveness of the measurement model was supported.

**TABLE 2:** Confirmatory factor analyses.

Model	χ^2^	*df*	*p*	CFI	RMSEA	90% CI	SRMR
Four-factor model	677	293	<0.001	0.901	0.067	0.060, 0.074	0.058
Three-factor model (Combing AS and PSS)	747	272	<0.001	0.869	0.855	0.071, 0.084	0.070
Two-factor model (Combing AS, PSS and OC)	1380	251	<0.001	0.660	0.125	0.119, 0.131	0.103
Single-factor model	1571	230	<0.001	0.142	0.067	0.136, 0.149	0.121

χ^2^, *chi-square statistic, CFI, comparative fit index, RMSEA, root mean square error of approximation, 90% CI refers to 90% confidence intervals for the RMSEA values; SRMR, standardized root means square residual.*

### Hypothesis Testing

Mediation results are displayed in Table 3. We used hierarchical regression to test H1 and H2. In the first step, we entered gender, age, education level, and organizational tenure as control variables into the regression equation (organizational commitment as the dependent variable). In the second step, we entered narcissism into the regression equation to test the negative predictive effect of narcissism on organizational commitment (b = –0.15, standard error [se] = 0.07, *t* = –2.06, *p* = 0.04). In the third step, we entered PSS into the equation (*b* = 0.51, se = 0.06, *t* = 8.287, *p* < 0.001). The results showed that the effect of supervisor support on organizational commitment was significant, while the predictive effect of narcissism on organizational commitment decreased and failed to reach significance (*b* = –0.06, se = 0.07, *t* = –0.92, *p* = 0.36). Therefore, hypotheses H1 and H2 were supported.

**TABLE 3:** Results of hierarchical regression analyses.

Organizational commitment	Step 1 b(se)	Step 2 b(se)	Step 3 b(se)
Constant	3.05(0.36) ***	3.50(0.42) ***	1.62(0.44) ***
Gender	–0.1(0.01)	–0.18(0.12)	–0.18(0.11)
Age	0.01(0.01)	0.00(0.01)	0.00(0.01)
Education	0.05(0.08)	0.07(0.08)	0.08(0.07)
Tenure	0.04(0.02)*	0.04(0.02)*	0.04(0.02)*
Narcissism		–0.15(0.07)*	–0.06(0.07)
Perceived supervisor support			0.51(0.06)***
R^2^	0.054**	0.068**	0.251***
R^2^ change		0.014*	0.183***

Unstandardized coefficients are reported. Numbers in parentheses are standard errors. *p < 0.05, **p < 0.01, ***p < 0.001.

**FIGURE 2:**
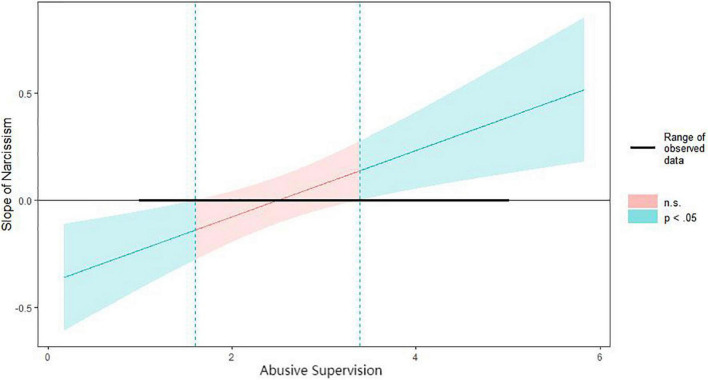
Johnson-Neyman interaction plots for abusive supervision and narcissism.

We used 5,000 bootstrap samples to construct 95% bias-corrected CIs to examine the moderation and moderated mediation effects via the PROCESS [model 7; Hayes (2013)] in R 4.1.0. As shown in Table 4, the interaction of narcissism and abusive supervision had a significant effect on perceived individual organizational support (*b* = 0.16, se = 0.059, 95% bootstrap CI = [0.018, 0.250]). This result supports H3a, and we used a Johnson–Neyman plot to identify better the interaction mode between narcissism and abusive supervision (Hayes and Montoya, 2017). As shown in Figure 2, except for interval [1.60, 3.39], the interaction between abusive supervision and narcissism was significant, and different moderation directions were shown at different degrees of abusive supervision. Therefore, H3a was further verified. At the same time, by further testing the significance of the indirect effect of abusive supervision in the path via which narcissism affects organizational commitment through supervisor support, the moderated mediation effect was supported. The index of moderated mediation was significant: b = 0.08, se = 0.03, 95% bootstrap CI = [0.009, 0.134]. Therefore, H3b was supported.

**TABLE 4:** Results of moderation effects analysis of abusive supervision using process.

Path	Effect	Boot SE	LLCI	ULCI
NA*AS→PSS	0.155	0.059	0.018	0.250
NA→PSS→OC	0.081	0.031	0.009	0.134

## Discussion

This study explored the impact of narcissism on employees’ organizational commitment and the role of PSS and abusive supervisors in this process. Through three waves of data collection, we confirmed that PSS is a vital mediator for the impact of narcissism on organizational commitment. At the same time, different degrees of abusive supervision have different effects on the supervisor support perceived by narcissistic employees.

### Theoretical Implications

First, this study enriches the literature on the relationship between employee personality factors and related outcome variables in the workplace. There is no consistent conclusion on the relationship between narcissism and organizational commitment in the existing literature (Michel and Bowling, 2013; Choi, 2019). Michel and Bowling (2013) proposed a positive relationship between narcissism and work attitude (job satisfaction and organizational commitment). They believe that narcissism positively correlates with work attitude due to narcissism’s inflated self-view, sense of self-superiority (Campbell et al., 2010), and perception of work mobility (Mcelroy et al., 1996). This study draws a conclusion that is contrary to it. We believe that the overestimation of one’s ability and the estimation of job-hopping ease may improve the individual’s job satisfaction. However, it will not enhance the individual’s psychological attachment to the current organization (Johnston et al., 1990) and thus enhance the sense of commitment to the organization. Similar to the conclusion of Choi (2019) study, narcissists are more sensitive to the perception of resource acquisition and consumption (Zhou et al., 2020), so they are more likely to be dissatisfied with the resource support from the organization and thus have a lower organizational commitment (Choi, 2019). This study supports the conclusion, extends the supply of organizational resources to the leadership level, and confirms that the resource support from supervisors can also impact the organizational commitment of narcissists. Narcissists possess personal charm and may also have destructive effects on the organization, Campbell et al. (2010) proved the importance of studying narcissistic personalities in organizational research. This study is a valuable supplement to exploring the influence of narcissistic personality on work attitude and its mechanism and boundary conditions and expands the depth of the study of narcissism in organizational backgrounds.

Second, this study discusses the influence of narcissistic personality on employees’ organizational commitment via PSS. We innovatively used COR theory to explain its internal psychological mechanism reasonably, which is beneficial to applying COR theory in organizations and further expands the application of COR theory in organizational research. Similar to Choi (2019) perspective, narcissism is shown not only in the individual’s cognition of self-ability, intimate relationships, and sense of self-superiority but also in the unique cognition of external resources (Zhou et al., 2020). Based on the relationship between individuals and external resources, COR theory discusses the possible effects of pressure caused by resources on individual cognition and behavior (Hobfoll et al., 2018). In this study, personality variables are brought into this theoretical framework. Individuals in the same situation may have different perceptions of environmental resources due to personality differences, resulting in different cognitive and behavioral consequences (Mischel and Shoda, 1995). This conclusion is helpful for us to understand the personality characteristics of narcissism from the perspective of resource conservation.

Finally, this study discusses the defensive psychological mechanism of narcissism against threats to self-value from the perspective of cognitive dissonance. Individual perception of environmental resource support is affected by their personality characteristics (Eaton and Bradley, 2008). The interaction between personality and environment will also affect the individual perception of environmental resource support. Personality has continuity and, at the same time, shows significantly different cognitive and behavioral characteristics at both ends of personality scores (Costa and McCrae, 1992). The maintenance of self-value constitutes the core personality characteristic of narcissism, so narcissists are extremely sensitive to external threats to self-value (Campbell et al., 2010). This study confirms that under the threat of high self-value, high narcissists do not decrease their perception of support from stressors as the general group does. Narcissistic employees will choose to actively change their cognition and carry out adaptive defensive strategies when facing environmental pressures that threaten their self-value, which is consistent with the basic view of cognitive dissonance theory (Harmon-Jones and Mills, 2019) and further deepen research on the cognitive style of narcissistic employees.

### Practical Implications

From the perspective of organizational practice, with increasing levels of narcissism in the general population (Hamamura, 2017), the issue of how to avoid the adverse impact of narcissism on an organization while giving full play to the positive aspects of narcissism is very important for organizational development (Campbell et al., 2010). Recognizing the negative impact of narcissism on employees’ organizational behavior and emotions, Campbell et al. (2010) suggested that organizational managers should improve their ability to identify narcissism in recruitment activities. However, although a large number of studies have confirmed the negative impact of narcissism on organizational development [e.g., Grijalva and Newman (2015) and Hamstra et al. (2021)], some scholars have pointed out that appropriate management practices for narcissists can enhance their initiative to a certain extent (Falco et al., 2020; Mao et al., 2021). This study demonstrates the impact of supervisor support on the relationship between narcissism and organizational commitment, which also implies that resource support in the organization is an effective way to improve the emotion of narcissistic employees toward the organization (Choi, 2019). On the other hand, Modrak et al. (2014) put forward the importance of appropriate “centralization” or “decentralization” for business process improvement. Given narcissistic employees’ desire for power and admiration in the organization (Campbell et al., 2010), we think it may be appropriate to include narcissists in the decision-making process through “decentralization.” The “centralization” or “decentralization” measures taken by the organization depend on many factors (Modrak et al., 2014), and bringing employees’ personality traits into this reference framework is conducive to the further refinement of organizational management.

In the face of the objective fact that the narcissistic population is increasing (Hamamura, 2017), we believe that blindly avoiding high narcissists from entering the organization is not an effective way to solve the problem. Explore the psychological mechanism by which narcissists produce negative behaviors and emotions, and giving full play to the bright side of narcissism on this basis is what an effective management system should do (Mao et al., 2021). The realization of this goal also calls for researchers to conduct a more detailed study of narcissism in the organizational context.

### Limitations and Future Research

We hope this study will shed some light on the literature interested in narcissistic employees’ cognitive and emotional patterns in an organizational context. However, although some valuable conclusions have been obtained, some shortcomings are still to be improved. First, although this study focused on general narcissism, the concept of narcissism can be further divided into different types (e.g., grandiose and vulnerable narcissism), and these types can be further subdivided and researched in-depth (Fang et al., 2021b). Although different narcissistic subtypes may have some commonalities in interpersonal relationships, they also involve different cognitive or behavior patterns (Miller et al., 2011). Therefore, future research can choose different types or dimensions of narcissism to improve the depth of research and further understand the cognitive performance and internal mechanism of narcissistic employees in the organizational context.

Second, situational measurement scales have superior psychometric characteristics to non-situational measures (Shaffer and Postlethwaite, 2012). However, the measurement of narcissism in the existing literature mostly depends on the general personality measurement [e.g., NPI: narcissistic personality inventory; Raskin and Terry (1988)], and there is a lack of tools to measure individual narcissism in the work situation. This study’s measure of employees’ narcissism comes from revising a measure of leadership narcissism in organizational situations (Hochwarter and Thompson, 2012). There is still no publicly available version of the narcissism scale for organizational employees. Therefore, future research needs to develop psychometrically sound and efficient measurement tools for narcissism with employees in the organizational context to improve the precision and scientific level of the research.

Finally, although this study is a valuable supplement to the employee narcissism literature, there is still a lack of effective interventions to reduce the adverse effects of narcissism on organizational commitment. Most studies propose to improve the ability to identify narcissistic personalities as much as possible in personnel recruitment. However, considering the positive significance of narcissism to the positive characteristics of leaders (Grijalva et al., 2014), blindly suppressing employee narcissism may also be detrimental to the organization’s development. Recognize that the adverse effects of narcissism on organizational commitment put forward higher management requirements for the organization. Maximizing the benefits of narcissism to the organization while reducing its adverse effects requires the joint efforts of more researchers in the future.

## Conclusion

Our study found that narcissism negatively affects employees’ organizational commitment and perceived supervisor support in organizational situations, and perceived supervisor support plays a mediation role in the relationship between narcissism and organizational commitment. At the same time, we find that due to the unique personality characteristics of narcissists, there is an interesting interaction between narcissism and abusive supervision behavior in the organization, and the supervisor support perceived by narcissists will increase in the face of abusive supervision. The research makes a unique contribution to our further understanding of narcissism and the role of narcissism in the organization.

## Data Availability Statement

The original contributions presented in the study are included in the article/supplementary material, further inquiries can be directed to the corresponding author.

## Ethics Statement

Ethical review and approval was not required for the study on human participants in accordance with the local legislation and institutional requirements. Written informed consent for participation was not required for this study in accordance with the national legislation and the institutional requirements.

## Author Contributions

All authors listed have made a substantial, direct, and intellectual contribution to the work, and approved it for publication.

## Conflict of Interest

The authors declare that the research was conducted in the absence of any commercial or financial relationships that could be construed as a potential conflict of interest.

## Publisher’s Note

All claims expressed in this article are solely those of the authors and do not necessarily represent those of their affiliated organizations, or those of the publisher, the editors and the reviewers. Any product that may be evaluated in this article, or claim that may be made by its manufacturer, is not guaranteed or endorsed by the publisher.

## Footnotes

1
https://wj.qq.com
